# Importance of anatomic variations in secundum ASD: residual ASD detected on postoperative 31st year

**DOI:** 10.1186/1749-8090-10-S1-A225

**Published:** 2015-12-16

**Authors:** Ufuk Yetkin, Nihan Karakaş, Mehmet Balkanay, Ali Gürbüz

**Affiliations:** 1 Department of Cardiovascular Surgery, Katip Celebi University Izmir Ataturk Training and Research Hospital, Izmir, Turkey

## Background/Introduction

Secundum ASD is the most common congenital defect seen in adulthood. Anatomical variations are the most important factors in decision making of surgical procedure.

## Aims/Objectives

Our case was 57 year-old male. He underwent secundum ASD repair 31 years ago. His complaint was progressive dyspnoea and for about 1 year.

## Method

Transthoracic echocardiography revealed residual ASD and transosophageal echocardiography showed that this ASD is a patch tear close to the orifice of inferior vena cava in size of 15x6 mm. Coronary angiography and cardiac catherization were performed and results were normal. Cavity sizes and pulmonary artery pressure were normal.

## Results

He was taken to operation, pericardium was very adherent and epicardium was cleaned with gentle dissection. Inferior vena cava cannulation was performed and the cannula was misguided to left atrium transseptally, it was detected by the help of blood color and cannula was redirected to IVC by special stretching manuevre. Under total cardiopulmonary bypass fossa ovalis type secundum ASD was explored and there was no patch component. An untouched sinus venous type ASD in size of 10X15 mm near IVC was detected and repaired with prolen sutures supported with teflon pledget. Successful decanulation and hemostasis after optimal dearing and residual ASD check, operation was terminated. No early or late complication was seen postoperatively and TEE showed no residual ASD.

## Discussion/Conclusion

We suggest that secundum ASD should be evaluated carefully especially by young surgeons as it may have anatomical variations and different localizations.

## Consent

Written informed consent was obtained from the patient for publication of this Case report and any accompanying images. A copy of the written consent is available for review by the Editor-in-Chief of this journal.

